# Follow-up care delivery in community-based hypertension and type 2 diabetes management: a multi-centre, survey study among rural primary care physicians in China

**DOI:** 10.1186/s12875-021-01564-z

**Published:** 2021-11-13

**Authors:** Yi Wang, Xiu-Jing Hu, Harry H. X. Wang, Hong-Yan Duan, Ying Chen, Yu-Ting Li, Zi-Lin Luo, Xin Li, Jia-Ji Wang, Stewart W. Mercer

**Affiliations:** 1 School of Public Health, Sun Yat-Sen University, No.74 Zhongshan Road 2, Guangzhou, 510080 People’s Republic of China; 2 The Center for Disease Control and Prevention of Zhejiang Province, Hangzhou, People’s Republic of China; 3 JC School of Public Health and Primary Care, Faculty of Medicine, The Chinese University of Hong Kong, Shatin, Hong Kong SAR; 4 Department of General Practice, Henan Provincial People’s Hospital, Zhengzhou, People’s Republic of China; 5 School of Public Health, Kunming Medical University, Kunming, People’s Republic of China; 6 State Key Laboratory of Ophthalmology, Zhongshan Ophthalmic Center, Sun Yat-Sen University, Guangzhou, People’s Republic of China; 7 Office of Cancer Screening, National Cancer Center/National Clinical Research Center for Cancer/Cancer Hospital, Chinese Academy of Medical Sciences and Peking Union Medical College, Beijing, People’s Republic of China; 8 Zhongshan School of Medicine, Sun Yat-Sen University, Guangzhou, People’s Republic of China; 9 Guangdong-Provincial Primary Healthcare Association, Guangdong, People’s Republic of China; 10 School of Public Health, Guangzhou Medical University, Guangzhou, People’s Republic of China; 11 Centre for Population Health Sciences, Usher Institute, University of Edinburgh, Edinburgh, Scotland, UK

**Keywords:** Follow-up care delivery, Hypertension, Type 2 diabetes, Treatment goal, Target non-attainment, Rural area, Primary care physicians

## Abstract

**Background:**

Follow-up care is crucial but challenging for disease management particularly in rural areas with limited healthcare resources and clinical capacity, yet few studies have been conducted from the perspective of rural primary care physicians (PCPs). We assessed the frequency of follow-up care delivered by rural PCPs for hypertension and type 2 diabetes – the two most common long-term conditions.

**Methods:**

We conducted a multi-centre, self-administered survey study built upon existing general practice course programmes for rural PCPs in four provinces. Information on follow-up care delivery were collected from rural PCPs attending centralised in-class teaching sessions using a set of close-ended, multiple choice questions. Binary logistic regression analysis was performed to examine physician-level factors associated with non-attainment of the target frequency of follow-up care for hypertension and type 2 diabetes, respectively. The final sample consisted of rural PCPs from 52 township-level regions. The Complex Samples module was used in the statistical analysis to account for the multistage sample design.

**Results:**

The overall response rate was 91.4%. Around one fifth of PCPs in rural practices did not achieve the target frequency of follow-up care delivery (18.7% for hypertension; 21.6% for type 2 diabetes). Higher education level of physicians, increased volume of daily patients seen, and no provision of home visits were risk factors for non-attainment of the target frequency of follow-up care for both conditions. Moreover, village physicians with less working experiences tended to have less frequent follow-up care delivery in type 2 diabetes management.

**Conclusions:**

Efforts that are solely devoted to enhancing rural physicians’ education may not directly translate into strong motivation and active commitment to service provision given the possible existence of clinical inertia and workload-related factors. Risk factors identified for target non-attainment in the follow-up care delivery may provide areas for capacity building programmes in rural primary care practice.

## Introduction

As the major preventable risk factors for cardiovascular disease (CVD) and premature death, hypertension and type 2 diabetes present long-lasting challenges to global public health as reflected by the enormous burden of morbidity and disability [1–3]. Along with the improved life expectancy and epidemiological transition, the rise in the number of adults with elevated blood pressure (BP) is now occurring largely in low and middle-income countries (LMICs) [4, 5]. Meanwhile, the prevalence of type 2 diabetes is also rising rapidly across LMICs, particularly in rural regions, and is associated with increased risk of all-cause mortality [6, 7].

Like many other developing countries, China is facing challenges with the awareness, treatment and control of long-term conditions such as hypertension and type 2 diabetes in rural areas [8, 9]. A highlight of national efforts to address healthcare gaps in China is the delivery of basic public health (BPH) service in primary care settings underpinned by government investment at both national and local levels to strengthen preventive health care [10, 11]. In rural regions, those who have completed nationally-accredited medical study curriculum at secondary education level or above are eligible for working as village physicians. The government statistics shows that most village physicians (93.4%) in China did not complete tertiary education (i.e., without a college or undergraduate degree) as of 2018 [12]. They work at village clinics and countryside infirmaries, serving as routine primary care physicians (PCPs) to deliver BPH preventive care in parallel with essential medical care in rural areas. The health management of hypertension and type 2 diabetes are free-of-charge items included as part of the BPH service package to enhance the capacity building for community-based disease prevention and health promotion that are responsive to community healthcare needs.

In the context of the nationwide BPH service provision, the follow-up care for hypertension and type 2 diabetes including general assessment of overall health, recommendations on lifestyles, and review of medical regime are indispensable for improving population health in primary care [13]. A substantial body of international evidence strongly suggests that primary care is one of the most cost-effective strategies for reducing morbidity, disability, and premature mortality attributed to long-term conditions [14, 15]. Meanwhile, existing literature also suggests that patient education and skill building could serve as facilitators to good patient adherence in hypertension and diabetes management for attaining the treatment goals [16, 17]. This would require enhanced patient-physician interactions built upon physicians’ capabilities to deliver a broad scope of person- and family-centred care to achieve desired health outcomes in the community. Rural areas, however, are likely to be subject to poor availability of healthcare resources and limited clinical capacity of village physicians. This may serve as a major obstacle to supporting regular delivery of community-based continuous care for rural populations.

A recommended frequency of 4 times per year for follow-up care delivery has been suggested in the recent Chinese national standards (3rd edition) for delivering BPH service in people diagnosed with hypertension or type 2 diabetes [18]. Nevertheless, little is known thus far about frontline physicians’ adherence to this recommended practice in the routine provision of rural primary care. International studies also demonstrate the possibility that education of rural health workforce does not always confer sustained effects in active commitment of physicians to rural community practice [19–21]. This would require more investigation and evidence. The main objective of this study was to assess the frequency of follow-up care delivered by rural PCPs for hypertension and type 2 diabetes – the two most commonly seen long-term conditions. We tested the hypothesis that physician-level factors, in particular the physician’s education level, were associated with non-attainment of the target frequency of follow-up care for both hypertension and type 2 diabetes in the study.

## Methods

### Study design

This was a multi-centre, survey study built upon existing course programmes on general practice (GP) education and training for rural primary care physicians (PCPs) in four provinces in China. In Yunnan and Guizhou provinces (western China), and Henan province (central China), a theoretical-practical training programme with centralised in-class teaching sessions was launched by the Chinese General Practice Young Professionals Alliance in 2019. This was partnered with the Chinese Medical Association to enrol PCPs in rural clinical practice for continuing medical education. Meanwhile in Guangdong province (southern China), a GP Professional Boost-up Training Programme was concurrently launched by the Guangdong Primary Healthcare Association (GDPHA) [22] – an officially registered body responsible for developing education and training that encompass the full scope of primary care. The Programme was established in conjunction with the Guangdong Health Commission to enhance the healthcare capacity of PCPs in rural areas where the Gross Domestic Product per capita falls below the national average.

### Setting and data source

The survey study was conducted on the sites where centralised in-class teaching sessions were held in each province. A set of close-ended, multiple choice questions drawn from literature review were used to gather self-report information from village physicians. The content validity of the survey was assessed by an expert panel consisting of two epidemiologists (YW and YC), two public health professionals (HHXW and YTL), and two GP consultants (HYD and JJW) who reviewed each item with regard to the relevancy and clarity. A pilot study was conducted among a systematic sample of 12 rural PCPs. The purpose of the project was introduced by the course instructor and questionnaires were disseminated to eligible class attendees by the on-site teaching assistant at the beginning of the course session. Participants were guided to return the anonymous, self-administered questionnaires to the course instructor during the session break. All the original questionnaires, upon the on-site check for completeness and correctness, were sent by postal mail to the study coordinating centre at Sun Yat-Sen University.

### Participants

We aimed to recruit at least 80% of rural PCPs fulfilling the eligibility criteria from centralised in-class teaching sessions, and a minimum of 120 PCPs were anticipated in each of the four provinces. The criteria of target subjects were those who 1) worked as rural primary care clinicians affiliated with village clinics or countryside infirmaries; 2) had class attendance on the day of data collection; and 3) practicing community-based follow-up care for hypertension and type 2 diabetes on a regular basis. Those who practiced chronic disease management in primary care for less than 12 months were excluded. The data collection was completed in August 2019 and the final sample consisted of rural PCPs from 52 township-level administrative regions.

### Study variables and measurements

We collected anonymous data on age, gender, ethnics, education level, years of GP working experiences, number of patients seen per day, physician-perceived healthcare needs, services delivered on chronic disease management, and settings and frequencies of follow-up care for hypertension and type 2 diabetes. In this study, we referred to the recent national standards for Basic Public Health Services (3rd edition) in China, where a frequency of 4 times per year for follow-up care delivery has been set as a recommended target in the hypertension and type 2 diabetes management [18].

### Statistical analysis

Two trained medical students independently entered the data with double entry verification in EpiData 3.1 (Denmark). Statistics with standard error (SE) or 95% confidence interval (CI), where appropriate, were applied in descriptive analysis. We conducted binary logistic regression analysis to examine physician-level factors associated with non-attainment of the target frequency of follow-up care for hypertension and type 2 diabetes, respectively, after controlling for confounders. A 20:1 rule was used for regression analysis where a minimum number of 400 participants was conservatively required for a regression model consisting of up to 20 independent predictor categories. A *p* value < 0.05 was considered statistically significant. All statistical analyses were done in IBM SPSS Statistics 25 (Chicago, IL, USA) and the Complex Samples module was used to account for the multistage sample design.

### Ethical consideration

Informed consent was obtained from all participants in the study. Data anonymisation was performed by removing subject identifiers from the dataset prior to data analysis. Ethics approval was granted from the School of Public Health Biomedical Research Ethics Review Committee at Sun Yat-Sen University (Ref: SYSUSPH2019032) in accordance with the Declaration of Helsinki 2013.

## Results

### Characteristics of survey participants

A total of 602 rural PCPs responded to the survey, with an overall response rate of 91.4%. No significant differences existed in the response rates at each study site. The mean age of survey respondents was 38.6 (SE 0.5) years, with one fifth of subjects aged 50 years and above. Male and female physicians accounted for an approximately equal proportion (51.5% vs 48.5%). More than one third (40.4%) of physicians were ethnic minorities. Slightly over half (53.3%) of participants had more than ten years of practicing primary care in rural areas. Nearly one third (30.6%) of survey participants had routine clinical encounters with over 20 patients per day. Over two thirds (71.8% [432/602]) of village physicians did not complete an undergraduate education. In general, survey participants with undergraduate education or above were younger (*p* < 0.001) and had shorter length of years in practicing primary care than their counterparts with lower education level (*p* < 0.001) **(**Table 1**)**.

**Table 1:** Characteristics of survey participants

Variables	Total (***N*** = 602)	Below undergraduate (***n*** = 432)	Undergraduate or above (***n*** = 170)	***P*** value
**Age, years**	38.6 (0.5)	39.6 (0.6)	35.9 (0.7)	< 0.001
**Age, groups**				
< 30 years	139 (23.1)	103 (23.8)	36 (21.2)	< 0.001
30–39 years	164 (27.2)	82 (19.0)	82 (48.2)	
40–49 years	178 (29.6)	144 (33.3)	34 (20.0)	
≥ 50 years	121 (20.1)	103 (23.8)	18 (10.6)	
**Gender**				
Male	310 (51.5)	231 (53.5)	79 (46.5)	0.01
Female	292 (48.5)	201 (46.5)	91 (53.5)	
**Ethnic group**				
Han Chinese	359 (59.6)	227 (52.5)	132 (77.6)	< 0.001
Minorities	243 (40.4)	205 (47.5)	38 (22.4)	
**Working experiences as rural PCPs**				
0–9 years	281 (46.7)	176 (40.7)	105 (61.8)	< 0.001
≥ 10 years	321 (53.3)	256 (59.3)	65 (38.2)	
**Number of patients seen per day**				
≤ 19	418 (69.4)	353 (81.7)	65 (38.2)	< 0.001
≥ 20	184 (30.6)	79 (18.3)	105 (61.8)	

Data are presented as n (%) or mean (SE) where appropriate. Chi-square tests or independent *t*-tests, where appropriate, were used to compare differences in age distribution, sex, ethnic group, working experiences, and number of patients seen per day between primary care physicians according to education level

*P* values larger than 0.01 were rounded to two decimal places

### Frequency and venue of follow-up care for hypertension and type 2 diabetes

Around one fifth of village physicians did not achieve the target frequency of follow-up care for hypertension and type 2 diabetes in the study. They reported a follow-up frequency of less than 4 times per year for hypertension (18.7%) and type 2 diabetes (21.6%), respectively. The majority of rural PCPs performed follow-up care through mixed clinic-based consultations and home visits, albeit a small proportion (17.6% for hypertension; 18.3% for type 2 diabetes) of follow-up care were delivered in clinic-based consultation rooms only **(**Table 2**)**.

**Table 2:** Provision of community-based follow-up care for hypertension and diabetes

Variables	N	% (95%CI)
**Provision of follow-up care for patients with hypertension**		
**Frequency of care delivery**		
Less than 4 times per year	112	18.7 (12.3 to 27.3)
4 times or above per year	490	81.3 (72.7 to 87.7)
**Venue of care delivery**		
Clinic-based consultation rooms only	106	17.6 (12.8 to 23.6)
Mixed clinic-based consultations and home visits	496	82.4 (76.4 to 87.2)
**Provision of follow-up care for patients with type 2 diabetes**		
**Frequency of care delivery**		
Less than 4 times per year	130	21.6 (15.5 to 29.3)
4 times or above per year	472	78.4 (70.7 to 84.5)
**Venue of care delivery**		
Clinic-based consultation rooms only	110	18.3 (13.6 to 24.2)
Mixed clinic-based consultations and home visits	492	81.7 (75.8 to 86.4)

*CI* confidence interval

### Physician’s perception of healthcare needs in follow-up care and routine practice

**Fig. 1:**
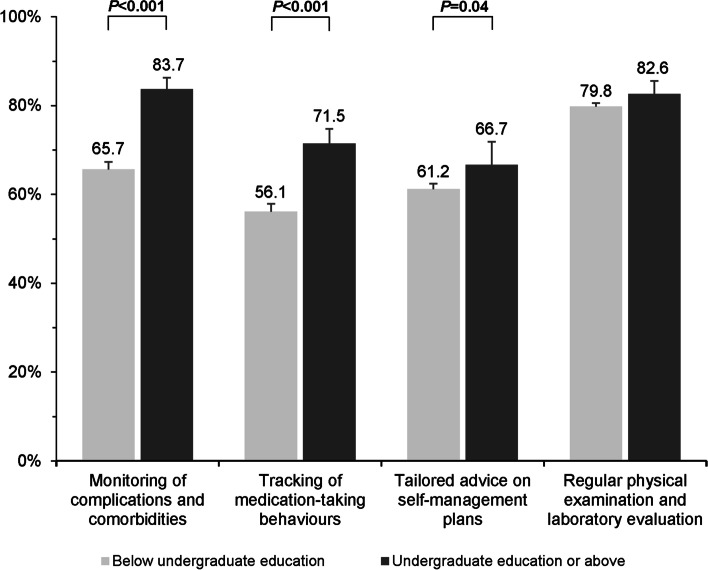
Physician-reported perception of individual healthcare needs in follow-up care for hypertension and type 2 diabetes by physician’s education level. Note: Error bars indicate 95% confidence intervals

**Fig. 2:**
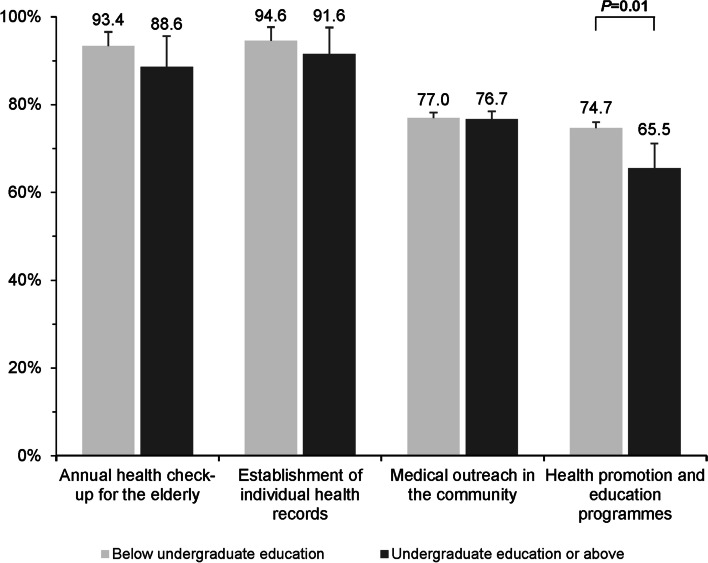
Physician-reported delivery of community-based management of hypertension and type 2 diabetes by physician’s education level. Note: Error bars indicate 95% confidence intervals

A significantly higher proportion of rural PCPs who recognised greater healthcare needs was observed among those having undergraduate education level or above when compared to their counterparts who had lower education level. These self-perceived healthcare needs included the monitoring of disease complications (83.7% vs 65.7%; *p* < 0.001), tracking of medication-taking behaviours (71.5% vs 56.1%; *p* < 0.001), and tailored advice given on self-management (66.7% vs 61.2%; *p* = 0.04) in follow-up care **(**Fig. 1**)**. In routine primary care practice, however, rural PCPs with higher education level tended to report less delivery of community-based activities, in particular health promotion and education programmes (65.5% vs 74.7%; *p* = 0.01) to manage hypertension and type 2 diabetes when compared to those with lower education level **(**Fig. 2**)**.

### Physician-level factors associated with target non-attainment in the follow-up care delivery

Rural PCPs with undergraduate education level or above (adjusted odds ratio [aOR] = 1.52, *p* = 0.049 for hypertension; aOR = 2.23, *p* = 0.001 for type 2 diabetes), having higher volume of patients seen per day (aOR = 4.23, *p* = 0.001 for hypertension; aOR = 2.33, *p* = 0.02 for type 2 diabetes), and who did not perform home visits as part of the service delivery (aOR = 4.13, *p* = 0.002 for hypertension; aOR = 3.20, *p* = 0.01 for type 2 diabetes) were more prone to be at risk for non-attainment of the target frequency of follow-up care delivery. Physicians with shorter lengths of time spent in rural primary care tended to practice less frequent follow-up care for type 2 diabetes (aOR = 1.75, *p* = 0.03), whilst such association was not significant for hypertension management **(**Table 3**)**.

**Table 3:** Logistic regression on physician-level factors associated with non-attainment of the target frequency of follow-up care delivery

	Model 1†		Model 2‡	
	aOR (95%CI)	***P***	aOR (95%CI)	***P***
Age, mean	1.00 (0.96, 1.04)	0.98	1.00 (0.97, 1.03)	0.89
Gender				
Male	1.00 (Ref)		1.00 (Ref)	
Female	1.10 (0.84, 1.44)	0.43	1.14 (0.80, 1.62)	0.43
Ethnic group				
Minorities	1.00 (Ref)		1.00 (Ref)	
Han Chinese	0.16 (0.10, 0.25)	< 0.001	0.57 (0.40, 0.81)	0.01
Education level				
Below undergraduate	1.00 (Ref)		1.00 (Ref)	
Undergraduate or above	1.52 (1.01, 2.29)	0.05	2.23 (1.55, 3.19)	0.001
Working experiences				
≥ 10 years	1.00 (Ref)		1.00 (Ref)	
0–9 years	1.67 (0.87, 3.20)	0.11	1.75 (1.09, 2.81)	0.03
Number of daily patients seen				
≤ 19	1.00 (Ref)		1.00 (Ref)	
≥ 20	4.23 (2.19, 8.16)	0.001	2.33 (1.23, 4.41)	0.02
Venue of follow-up delivery				
Mixed clinic and home visits	1.00 (Ref)		1.00 (Ref)	
Clinic consultation rooms only	4.13 (1.99, 8.58)	0.002	3.20 (1.59, 6.44)	0.01

*aOR* adjusted odds ratio; *CI* confidence interval

†Model 1: Dependent variable: frequency of follow-up care < 4 times per year for hypertension

‡Model 2: Dependent variable: frequency of follow-up care < 4 times per year for type 2 diabetes

*P* values larger than 0.01 were rounded to two decimal places

## Discussion

### Main findings

We found that according to this self-administered survey, around one fifth of PCPs in rural practices were unable to achieve a target frequency of 4 times per year for hypertensive and type 2 diabetic follow-up care delivery. When compared to village physicians with lower education level, those with higher education level perceived greater healthcare needs for follow-up care, but reported less community-based service delivery. Higher education level, increased daily patient volume, and no provision of home visits were physician-level risk factors associated with non-attainment of the target frequency of follow-up care for both conditions. In addition, village physicians with less working experiences tended to have less frequent follow-up care delivery in the diabetes management.

### Relationship with other studies

Follow-up care is of great importance to the management of long-term conditions such as hypertension and diabetes as patients often require ongoing treatment and continuous care. Nearly 40% of the total Chinese population live in rural areas as of 2018, accounting for the second largest proportion of the rural population of the world [23]. However, the growing rural-urban health inequalities have been documented in both developed and developing countries [24, 25]. People living in more deprived rural areas tend to face greater challenges from poor accessibility of healthcare services and suboptimal physician capacity than that in more urbanised regions as a result of the ‘inverse care law’ [26–28]. International experience has suggested an important role of village physicians in the delivery of community-based healthcare services as the major primary care provider in rural areas [29].

We found that more than two thirds of rural PCPs participated in the study did not have an undergraduate education, which is consistent with other studies [30–33]. While patient education has played a role in achieving better BP and glycaemic control [16, 17, 34], a lack of physician’s continuing medical training is one of the notable barriers to enhance capacity building. Previous research has raised concerns over the poor availability of qualified healthcare professionals in rural areas and the physician’s inherent pursuit of working opportunities in urban areas given the advanced medical technology, higher remuneration and better career prospect [35]. This may be particularly common among the ethnic minorities who often reside in more remote areas with relatively poor medical resources and high illiteracy rates [36, 37], and village physicians of this group were therefore less likely to achieve a target frequency of 4 times per year for follow-up care for hypertension and type 2 diabetes as shown in our study.

Previous documents have reported the inability or failure of physicians to initiate or intensify therapy when a more aggressive course is recommended by guidelines, known as ‘clinical inertia’ in routine practice [38]. This could exist in all stages of disease management, including the beginning of lifestyle changes and strengthening of treatment [39]. Interestingly, our findings showed a positive correlation between physician’s higher education level and perceived greater healthcare needs in follow-up care, which may be a result of proper knowledge and understanding of best practice acquired from better education. Nevertheless, the opposite was also illustrated in the correlation of physician’s education with self-reported care delivery in routine practice, implying that better education itself may not directly translate into strong motivation and active commitment to primary care service provision. One possible interpretation is that upon the completion of higher education, village physicians may envisage more professional autonomy such as clinical work freedom [40], thus practicing less community-based services although they were able to realise the greater healthcare needs for follow-up care.

The physician’s adherence to recommended clinical guidelines on follow-up care delivery may also be influenced by self-perceived workload. Workload characteristics such as the number of patients seen or administrative burdens have been reported to be associated with physician’s job satisfaction [30, 41, 42]. We found that village physicians with a higher volume of patients seen per day tended to have less frequent delivery of follow-up care, which were common for both hypertension and diabetes. Under the circumstances of increased clinic-based workload, the delivery of community-based continuous care could be shrunk as a result of physician burnout [43]. The reduced initiative and motivation due to additional workload may also explain the significant association between shorter lengths of working experiences and less frequent care delivery particularly in the follow-up care for diabetes. The blood glucose test for diagnosis and monitoring requires a blood-taking procedure, which may cause extra workload on top of the blood pressure measurement perceived by junior rural physicians who have not yet achieved clinical proficiency of handling complex encounters. This may warrant further qualitative investigations to determine the extent to which self-perceived workload impacts on daily practice among village physicians of this group.

Our results suggested that the delivery of home visits as part of follow-up care also played a role. It is believed that home visits can strengthen patient-physician relationship and help physicians understand patient’s culture and preferences, adding knowledge and insights to GP profession [44]. A home visit on top of routine care delivered at clinic consultation rooms is more likely to reach patients who are busy during office hour or those with disabilities, and thus physicians are more prone to achieve the recommended goal of follow-up frequency. This echoes existing literature on patient-reported barriers to routine follow-up care for hypertension and diabetes in low-income settings, including but not limited to transportation, financial burden and schedule conflicts, along with treatment adherence and satisfaction [45, 46]. Besides, it has been suggested that therapeutic-related factors could also be related with achieving optimal practices in disease management on top of health education [16, 17, 34]. For instance, combined anti-hypertensive treatment was found to be superior to treatment with single drug in achieving BP goals in subjects with hypertension [47]. Recent evidence shows that advanced tele-monitoring techniques such as home-based blood pressure monitoring are capable of improving medication compliance and reducing blood pressure, with minimum additional workload for physicians [48, 49]. This could offer novel options for promoting disease management at home on top of conventional approaches to address barriers to follow-up care, and thus broaden the scope of primary care practice to accommodate healthcare needs of the local community.

### Strengths and weakness of the study

Follow-up care is crucial for community-based hypertension and type 2 diabetes management particularly in low-resource settings, yet few studies have been conducted from the perspective of village physicians. We collected data from rural PCPs including ethnic minorities with a variety of geographic locations in southern, western and central China to increase the diversity of study subjects. A focus was placed on community-based follow-up care for the two conditions that are most prevalent health problems both nationally and globally. A Complex Sample design was accounted for in the analysis to improve statistically valid inferences. However, our results should be interpreted with caution. Firstly, as primary care providers are geographically dispersed across the vast expanse of rural areas, it is less feasible to visit each GP clinic for subject recruitment. Instead, study participants were approached in the setting of centralised in-class sessions where village physicians came to attend for continuing medical education through existing GP course programmes. As those who did not enrol in such programmes during the study period were not captured, it may affect the generalisability of our findings to the entire village physicians in China. Secondly, the reliance on physician’s self-report of follow-up care delivery may subject to recall bias due to the absence of available data retrieved from electronic health record system. Thirdly, confounders potentially associated with care delivery such as job satisfaction may not be fully adjusted for in this study, and a physician self-report survey will inevitably restrict inclusion of questions relating to individual characteristics at patient-level. Accordingly, we were unable to differentiate whether patients aren’t coming back out of their own volition versus because of the provider, despite the possibility that patient-level barriers such as transportation, financial burden and schedule conflicts might play a role [45, 46]. Fourthly, factors associated with target non-attainment in this study may not directly indicate its correlation with patient outcomes, and the use of a specific health-status measurement as the primary outcome from the patient’s perspective is warranted in future studies. Last but not least, a cause-and-effect relationship could not be established given the cross-sectional nature of the study. Future large-scale studies shall extend the coverage of study subjects to a wider group of rural PCPs and service users with the assistance of internet-based, longitudinal data collection based on computerised health record.

### Implications for clinical practice

Our findings could increase the understanding of follow-up care delivery among rural PCPs and inform areas for capacity building programmes targeted village physicians in rural primary care practice. It is worthy of note that patients who are at high risk of cardiovascular events may need more intensive follow-up care, and therefore the hindering factors identified in our study for achieving the recommended goal frequency of follow-up care may bear greater primary care challenges [50, 51]. International evidence has suggested that increased annual number of primary care visits could be associated with increased likelihood of improved longitudinal health outcomes, and may be related with less hospital admissions and decreased healthcare costs [52–54]. Efforts that are solely devoted to enhancing rural physicians’ education may not suffice for chronic care management given the possible co-existence of clinical inertia and workload-related factors. A mixed clinic and home visits is recommended for follow-up care delivery; nevertheless, this would inevitably require computer-aided telehealth capabilities, clinical decision-support tools and infrastructure support in the context of rural health-care resources. A recent real-world trial conducted at the county setting reported the effectiveness of a healthcare intervention comprising education and feedback for PCPs through an electronic decision support system in overcoming clinical inertia [55]. From a service delivery perspective, the barriers (or facilitators) such as service sites, the training of PCPs, clinical capabilities and physician involvement should be incorporated in the formulation of evidence-based health care strategies intended to optimise the implementation of clinical practice recommendations in rural areas with resource limitations.

## Conclusions

Physician-level factors were associated with the routine delivery of community-based, follow-up care for hypertension and type 2 diabetes in rural primary care settings in China. Physicians with higher education level perceived greater healthcare needs for follow-up care; however, they reported less delivery of community-based disease management activities. Higher education level, increased daily patient volume, and no provision of home visits served as risk factors associated with non-attainment of the target frequency of follow-up care for both hypertension and type 2 diabetes. Rural primary care physicians with these risk factors should be given particular attention in future GP development programmes to scale-up capacities in managing long-term conditions in rural areas.

## Data Availability

The data that support the findings of this study are available from the corresponding author upon reasonable request.
